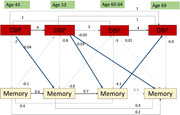# Bidirectional associations between blood pressure and memory decline over 25 years: a 1946 British Birth cohort population‐based study

**DOI:** 10.1002/alz.093388

**Published:** 2025-01-09

**Authors:** Sarah‐Naomi James

**Affiliations:** ^1^ UCL, London, England, United Kingdom; MRC Unit for Lifelong Health and Ageing at UCL, London, United Kingdom; Dementia Research Centre, UCL Queen Square Institute of Neurology, University College London, London United Kingdom

## Abstract

**Background:**

Midlife blood pressure is a risk factor for cognitive impairment. Yet, the directional relationship between blood pressure and memory may vary across adulthood, be confounded by earlier life factors, and vary by sex. Using a population‐based cohort of people born in the same week, we investigate the bidirectional associations between diastolic blood pressure (DBP) and memory, spanning over 25 years of adulthood.

**Method:**

Data from participants (n = 1922, 52% female) in the British 1946 birth cohort were included. DBP and Word Learning Test (WLT) performance were assessed at four points across adulthood (ages 43, 53, 60 and 69) by nurse visits. WLT incorporated three 15‐item word recall (summed to give a maximum score of 45) with alternating lists across assessments. Autoregressive cross‐lagged models assessed the bidirectional associations between WLT and DBP. Models were tested for sex effect modification and adjusted for childhood cognition; childhood socioeconomic position; education; and hypertensive medication use.

**Result:**

The autoregressive cross‐lagged models revealed an independent unidirectional relationship between higher DBP in midlife predicting lower subsequent cognition (i.e. higher DBP at age 43 predicting lower memory at age 53; higher DBP at age 53 predicting lower memory at age 60 and 69, additively) (Figure 1). In contrast, there was a unidirectional relationship between later‐life cognition (at age 60) predicting higher subsequent DBP (at age 69), with preliminary results demonstrating a stronger effect in women. In a sex‐adjusted model, a 1 SD higher memory score at age 60 was associated with 4mmMg increase in DBP at age 69. Adjusting for childhood cognition, childhood SEP and education largely attenuated these effects but results mainly remained significant at the 5% level.

**Conclusion:**

We provide evidence that higher midlife blood pressure is associated with subsequent lower memory, in line with previous studies, and even after adjustment for strong predictors of cognition, such as childhood cognition, education and SEP. However, our results also demonstrate evidence of a reverse association later in life, whereby lower memory is associated with subsequent lower blood pressure suggesting that cognitive processing may play a role in diastolic ageing processes, particularly in women.